# Comparison of three different image enhancement systems for detection of laryngeal lesions

**DOI:** 10.1017/S0022215123000981

**Published:** 2024-01

**Authors:** Fabian Kraus, Sven Gehrke, Desiree Ehrmann-Müller, Frank Hofer, Wafaa Shehata-Dieler, Rudolf Hagen, Agmal Scherzad

**Affiliations:** 1 Department of Oto-Rhino-Laryngology, Plastic, Aesthetic and Reconstructive Head and Neck Surgery, University of Würzburg, Würzburg, Germany; 2 School of Economics and Business Administration, University of Jena, Jena, Germany,; 3 Department of Oto-Rhino-Laryngology, Head and Neck Surgery, Bundeswehr Hospital Ulm, Ulm, Germany

**Keywords:** Larynx, hypopharynx, endoscopy, pathology, neoplasm, head and neck neoplasms

## Abstract

**Objective:**

Image enhancement systems are important diagnostic tools in the detection of laryngeal pathologies. This study aimed to compare three different image enhancement systems: professional image enhancement technology, Image1 S and narrow-band imaging.

**Method:**

Using the three systems, 100 patients with laryngeal lesions were investigated using a flexible and a 30° rigid endoscope. The lesions were diagnosed by three experts and classified using the Ni classification. The findings were compared.

**Results:**

Lesions classified as ‘benign’ were histopathologically confirmed in 50 per cent of patients, malignant lesions were confirmed in 41 per cent and recurrent respiratory papillomatosis were confirmed in 9 per cent. There was no significant difference between the experts’ assessments of each image enhancement system.

**Conclusion:**

The three systems give comparable results in the detection of laryngeal lesions. With two additional systems, more users can perform image-enhanced endoscopy, resulting in a broadly available tool that can help to improve oncological assessment.

## Introduction

The detection of epithelial and subepithelial vascular changes is essential in laryngeal and hypopharyngeal lesions, as such changes can indicate premalignancy and malignancy. Endoscopes with image enhancement systems can be used to detect conspicuous vascular patterns.^1–6^

Since the last decade, the first available image enhancement system, narrow-band imaging (Olympus Medical Systems, Tokyo, Japan), has been routinely used in otorhinolaryngology.^1–5,7^ Narrow-band light with wavelengths of 415 and 540 nm is reflected in the superficial and submucosal layers of tissue.

The Image1 S modular camera system (Karl Storz, Tuttlingen, Germany) and Xion's professional image enhancement technology (Xion Medical, Berlin, Germany) are newer digital systems that use an algorithm to amplify the colour contrast, shift the colour spectrum and increase the image definition. The raw image data are used to perform the picture analysis. The digital algorithm produces approximately the same change in contrast as illumination with narrow-band light.^3,8,9^

The main focus in these techniques is neoangiogenesis and visualisation of the vessels.^4^ A proliferation of vessels with dilated and submucosal capillary loops are connected in premalignant and malignant lesions. In particular, the intersections between a suspicious lesion and regular soft tissue are important.^10^ The literature shows significantly better differentiation of the vascular pattern with an image enhancement system in comparison to using white light.^1–7,11,12^

There are few data in the literature on the use of image enhancement techniques other than narrow-band imaging.^3,7–9^ Staníková *et al*. showed that Image1 S and narrow-band imaging are comparable.^3^ However, narrow-band imaging is an online technique using narrow-band light. Image1 S and professional image enhancement technology use raw data for digital amplification.

The detection of pathological vessels in pre-operative assessment as well as in post-operative monitoring is of great interest. So far, only narrow-band imaging is known to perform an ‘optical biopsy’.^3^ With two additional systems, more surgeons would have the option to use an amplification technique in oncology patients.

This study aimed to compare the accuracy of pre-operative and intra-operative diagnosis for three different image enhancement systems: professional image enhancement technology, Image1 S and narrow-band imaging. The pre-operative and intra-operative findings were subsequently correlated with histology findings.

## Materials and methods

This prospective study was approved by the responsible ethical committee (approval number: 2021020301) and conducted according to the guidelines established in the Declaration of Helsinki (Washington, 2002) for conducting clinical studies involving humans.

A total of 100 patients with a primary laryngeal lesion were investigated between October 2020 and May 2021. Patients with epithelial changes after laryngeal surgery, radiotherapy or radiochemotherapy were excluded. The group comprised 34 female and 66 male patients aged 21–84 years.

Transnasal endoscopy involving use of the professional image enhancement technology system followed by transnasal flexible endoscopy using the narrow-band imaging system was carried out 1–3 days before microlaryngoscopy. During the microlaryngoscopy under general anaesthesia procedure, endoscopy (with a 30° rigid endoscope) using the Image1 S system was performed prior to any surgical procedure (biopsy or resection). The laryngeal lesions were categorised according to the Ni classification.^10^ Afterwards the results of the endoscopy and the histopathological investigation were compared.

The endoscopy images were blinded and randomised before evaluation by three otolaryngologists who were not aware of the histology or diagnosis. The investigators had, respectively, 1, 4 and more than 10 years of experience with endoscopic image-enhanced evaluation of vascular patterns. The type of vascular architecture and the extent of microvascular patterns were compared between methods.

### Image enhancement techniques

The pre-operative flexible endoscopies were performed using a high-definition flexible video-nasopharyngoscope with the professional image enhancement technology system (Video Nasopharyngoscope XN HD; Xion Medical), and using a rhino-laryngo-videoscope with narrow-band imaging illumination (Flexible Video Endoscope ENT-VH; Olympus Medical Systems), in an upright sitting position. With both endoscopes, a white balance using a standard white balance tube was performed before use. The images were recorded with digital video archive software (‘DiVAS’; Xion Medical). During rigid endoscopy under general anaesthesia, evaluation was completed using the Image1 S system before any manipulation of the soft tissue. The images were recorded using the Aida system (Karl Storz).

### Professional image enhancement technology

Professional image enhancement technology uses a digital algorithm to amplify colour contrast, shift the colour spectrum and increase the image definition. In order to obtain better differentiation of the tissue, the red colour spectrum is amplified (based on information from Xion Medical).

### Narrow-band imaging

The optical filter of narrow-band imaging allows penetration of narrow-band light with wavelengths of 415 nm (blue light) and 540 nm (green light). Depending on the soft tissue, the narrow-band imaging light is absorbed in different ways, leading to a higher contrast between vessels and the surrounding mucosa.

### Image1 S illumination algorithm

Based on a software algorithm (‘Clara’), Image1 S provides images with homogeneous illumination as it dynamically brightens up dark areas in the background. By modulating the green, blue and red channels, points of interest are highlighted (based on information from Karl Storz).

### Classification of vascular patterns

The evaluation of vascular patterns was carried out using the three different recordings made with professional image enhancement technology, Image1 S and narrow-band imaging. The analysis followed the laryngeal lesion classification of Ni *et al*.^10^ Ni *et al*. considered types I–III as non-malignant, type IV as precancerous lesion-like recurrent respiratory papillomatosis and type V as malignant with an overall sensitivity of 88.9 per cent and specificity of 93.2 per cent. Types I–III were therefore merged into one ‘benign’ category and types IV and V into one ‘malignant’ category in this study.

### Statistical analysis

The measured values were statistically examined with two intentions. The first research question analysed three experts’ assessments of the three different image enhancement systems and examined differences between the experts. This analysis used Fleiss’ kappa, which is a multi-rater extension of the well-known Cohen's kappa for inter- and intra-rater comparison. In contrast to the percentage agreement, the Cohen/Fleiss kappa calculation takes random matches into account.

The second analysis addressed how well the three image enhancement systems could predict malignant characteristics. As the Ni laryngeal lesion classifications are not directly transferable into the benign/premalignant/malignant pathological system, the following assumptions were made: (1) Ni classifications I–III were considered ‘benign’; (2) Ni classifications IV and V were considered ‘malignant’; and (3) ‘premalignant’ histological cases were classified as ‘malignant’.

The three experts evaluated the samples independently. As the values fall into two categories, we applied the chi-square test for independence as well as McNemar's *t*-test. Based on the cross-tabulation, the sensitivity (true positive rate) and specificity (true negative rate) were calculated with the malignant cases as the positive class.

## Results and analysis

**Figure 1.**
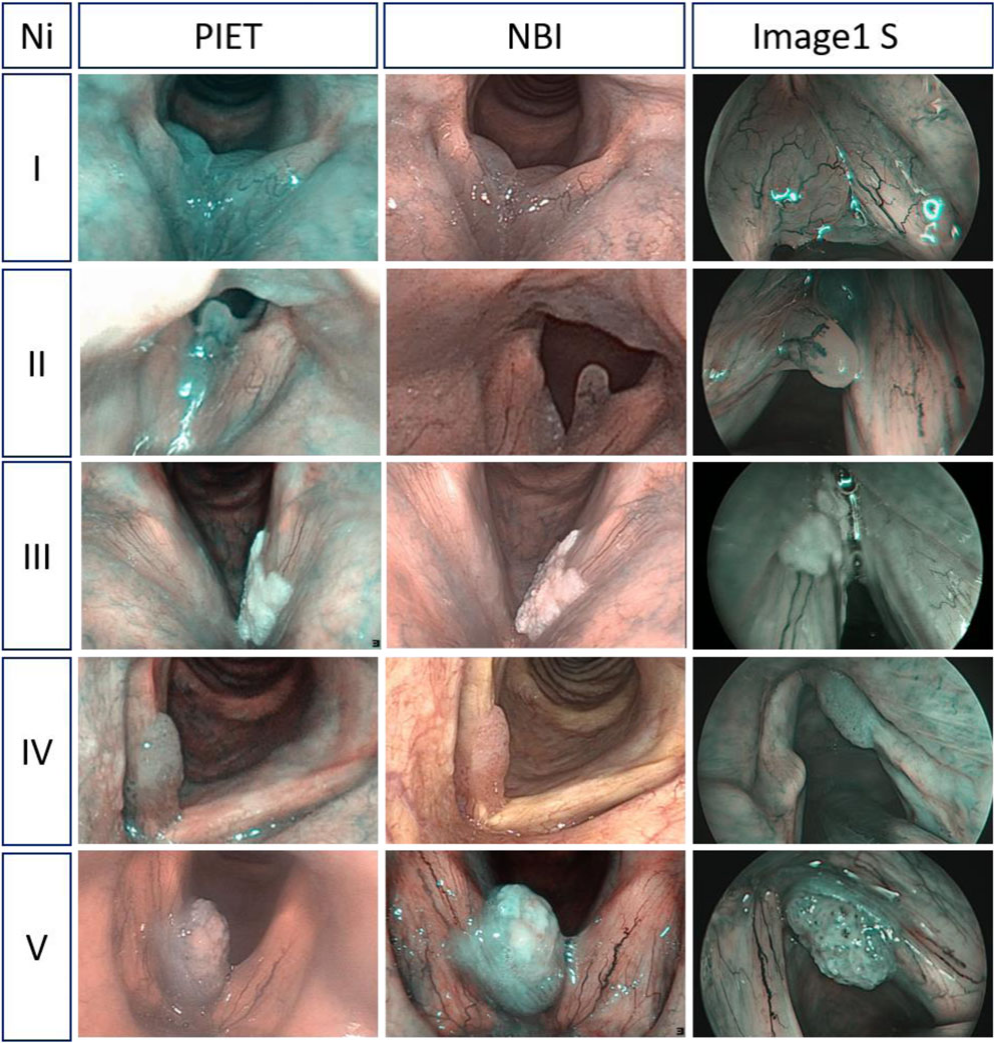
Comparison of flexible endoscopy with professional image enhancement technology (PIET), narrow-band imaging (NBI) and Image1 S regarding Ni classifications of laryngeal lesions with different clinical findings.

Between October 2020 and May 2021, 100 patients (34 females and 66 males; age range 21–84 years, average age of 60.5 years) with a primary laryngeal lesion who were receiving microlaryngoscopy were identified. Benign lesions were confirmed histopathologically in 50 cases (50 per cent), malignant lesions in 41 cases (41 per cent) and recurrent respiratory papillomatosis as premalignant lesions were confirmed in 9 cases (9 per cent). The histopathological examination confirmed benign lesions (polyp, cyst, Reinke's oedema, granuloma, hyperplastic verruca, chronic inflammation) in 50 per cent of cases, recurrent respiratory papillomatosis in 9 per cent and malignant lesions (severe dysplasia, carcinoma in situ, invasive carcinoma) in 41 per cent (Figure 1).

### Image enhancement with professional image enhancement technology

With professional image enhancement technology, rater 1 classified Ni types I–III (benign) in 47 per cent of cases, rater 2 in 52 per cent and rater 3 in 50 per cent. Ni type IV (recurrent respiratory papillomatosis or potential precancerous lesion) was classified in 29 per cent (rater 1) and 23 per cent (raters 2 and 3) of cases. Vascular changes associated with Ni type V were detected in 24 per cent of cases by rater 1, in 25 per cent by rater 2 and in 27 per cent by rater 3.

Overall, the optical rating ‘benign’ was confirmed histologically in 143 cases. In six ratings, a malignant lesion was histopathologically detected. Of cases rated as malignant, 144 specimens were verified as malignant tissue on histology. In seven cases, the malignant rating was not confirmed and the specimens were found to be benign (Table 1).

**Table 1.** Comparison of endoscopic imaging and histopathological findings

Optical biopsy finding	PIET		Narrow-band imaging		Image1 S	
	Histology finding		Histology finding		Histology finding	
	Benign	Malignant	Benign	Malignant	Benign	Malignant
Benign	143	6	141	5	135	3
Malignant	7	144	9	145	15	147

Data represent numbers of cases. PIET = professional image enhancement technology

### Image enhancement with narrow-band imaging

Using narrow-band imaging, rater 1 classified 47 per cent of cases as Ni types I–III, 25 per cent as type IV and 28 per cent as type V. Rater 2 diagnosed 50 per cent of cases as Ni types I–III, 26 per cent as type IV and 24 per cent as type V. Rater 3 classified 49 per cent of cases as Ni types I–III, 22 per cent as type IV and 29 per cent as type V. A benign rating with narrow-band imaging was confirmed in 141 cases. Five specimens postulated as being non-malignant showed malignancy in the histopathological evaluation, 145 samples were confirmed as malignant and 9 cases with a malignant rating were found to be benign on histology (Table 1).

### Image enhancement with Image1 S

Image enhancement during 30° rigid endoscopy under general anaesthesia was defined by rater 1 as Ni types I–III in 43 per cent of cases, as type IV in 16 per cent and as type V in 41 per cent. Rater 2 classified 47 per cent as Ni types I–III, 17 per cent as type IV and 36 per cent as type V. Finally, rater 3 categorised Ni types I–III in 48 per cent, type IV in 16 per cent and type V in 36 per cent. The optical ‘non-malignant’ rating was confirmed in 135 cases. Three specimens with a non-malignant rating showed malignancy on histopathological examination and 147 specimens rated as malignant were verified as being malignant tissue on histology. In 15 cases, the malignant rating was not confirmed (Table 1).

### Statistical analysis

The results of the flexible professional image enhancement technology and narrow-band imaging examinations in a sitting position and the Image1 S evaluation with rigid endoscopy under general anaesthesia were statistically analysed to determine the histological agreement for each system and the agreement of the three different raters, each with different experience of endoscopic image-enhanced evaluation of vascular patterns.

### Professional image enhancement technology and histology

The correlation between histology and professional image enhancement technology showed an accuracy of 0.95 (*κ* = 0.91, 95 per cent confidence interval (CI) 0.927–0.976, *p* < 0.01, McNemar's *t*-test). For malignancy detection, the sensitivity was 0.96 with a specificity of 0.95. The chi-square test confirmed these results (χ^2^ = 246.62, *p* < 0.001).

### Narrow-band imaging and histology

The correlation between histology and narrow-band imaging showed an accuracy of 0.953 (*κ* = 0.90, 95 per cent CI = 0.922–0.974, *p* < 0.01, McNemar's *t*-test). For malignancy detection, the sensitivity was 0.96 with a specificity of 0.94. The chi-square test confirmed these results (χ^2^ = 243.17, *p* < 0.001).

### Image1 S and histology

The correlation between histology and Image1 S showed an accuracy of 0.94 (*κ* = 0.88, 95 per cent CI = 0.906–0.964, *p* < 0.01, McNemar's *t*-test). For malignancy detection, the sensitivity was 0.98 with a specificity of 0.90. The chi-square test confirmed these results (χ^2^ = 230.29, *p* < 0.001).

### Image enhancement and Ni I–V

**Figure 2.**
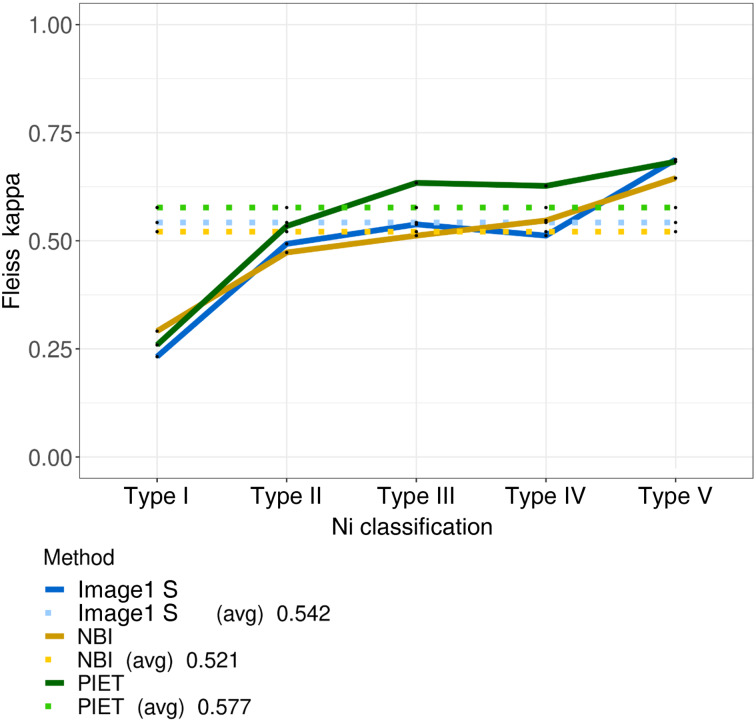
Agreement (Fleiss’ kappa) between the three different image enhancement systems in terms of Ni laryngeal lesion classification (types I–V). avg = average; NBI = narrow-band imaging; PIET = professional image enhancement technology

The statistical analysis of the different image enhancement systems for the five Ni classifications of laryngeal lesions showed a stronger agreement for higher Ni types (IV and V). The average Fleiss kappa index was 0.577 for professional image enhancement technology, 0.521 for narrow-band imaging and 0.542 for Image1 S (Figure 2).

### Recurrent respiratory papillomatosis

In recurrent respiratory papillomatosis, a typical vascular pattern can be detected. All nine patients with recurrent respiratory papillomatosis were diagnosed using the three different image enhancement systems by all three raters.

### Experience in diagnosis using image enhancement

**Figure 3.**
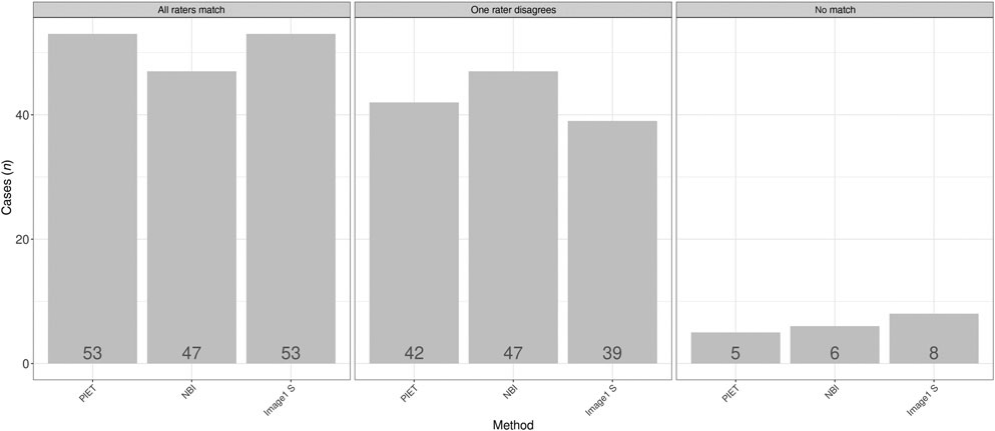
Differences according to image enhancement method for the three raters. PIET = professional image enhancement technology; NBI = narrow-band imaging

The diagnosis of vascular changes requires consistent evaluation of the image-enhanced pictures. The three investigators had, respectively, 1, 4 and more than 10 years of experience with endoscopic image-enhanced evaluation of vascular patterns. The assessments by the different raters using the professional image enhancement technology system showed 53 per cent agreement between them. For 42 per cent of cases, two raters gave the same Ni laryngeal lesion classification. In 5 per cent of cases, the three raters attributed a different Ni classification (Figure 3).

**Figure 4.**
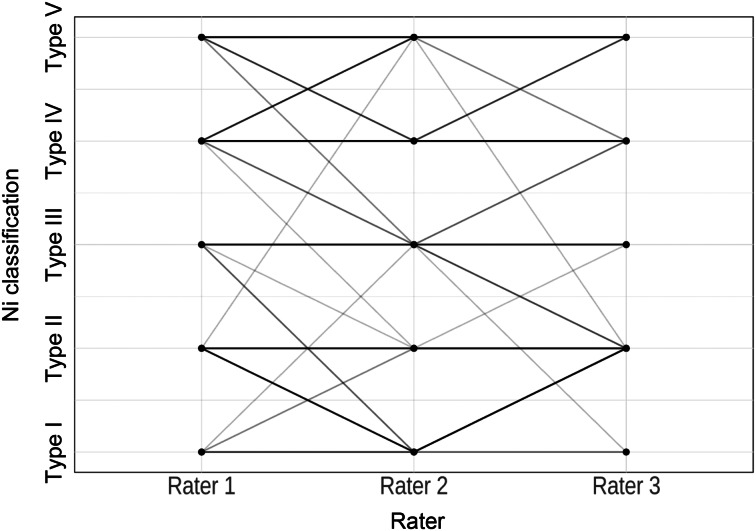
Parallel co-ordinate plot comparing professional image enhancement technology examination for raters 1, 2 and 3.

**Figure 5.**
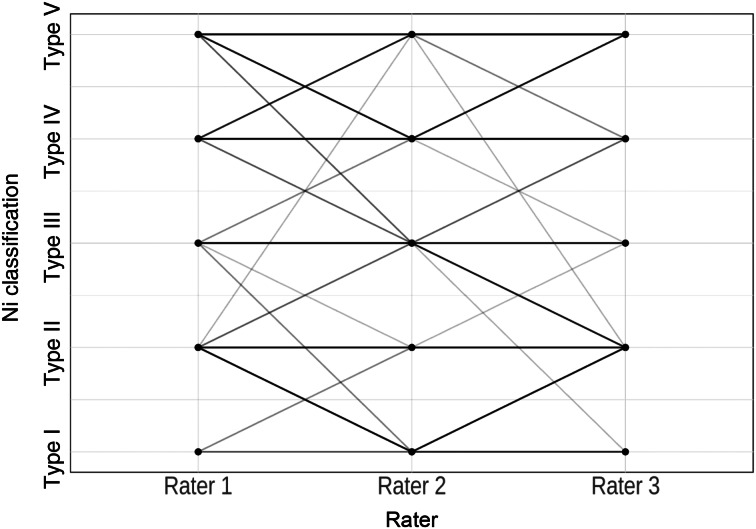
Parallel co-ordinate plot comparing narrow-band imaging examination for raters 1, 2 and 3.

**Figure 6.**
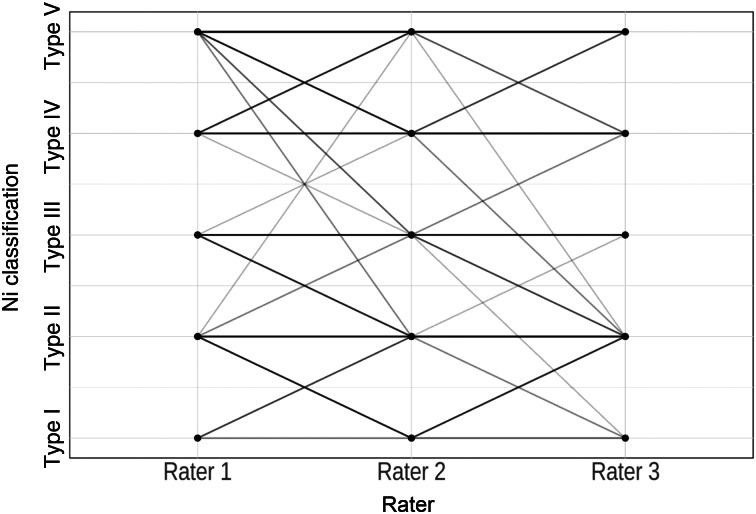
Parallel co-ordinate plot comparing Image1 S examination for raters 1, 2 and 3.

The parallel co-ordinate plots display a high level of agreement between the raters, as indicated by the parallel lines (Figures 4–6): the more cases of agreement, the darker the lines. The visualisation of the data using a parallel co-ordinate plot illustrates good differentiation between the lower Ni lesion types I–III (benign) and the higher Ni lesion types IV and V (malignancy).

## Discussion

Early diagnosis of malignant changes can help to improve oncological treatment.^7,12^ In recent decades, endoscopic evaluation of vascular patterns has enabled early detection of cancerous lesions.^4,5,8^ A standardised and structured examination of vascular changes is essential for prognostic information and to plan therapeutic strategies. In the literature, narrow-band imaging and Image1 S have been evaluated for use in endoscopic procedures.^1,2,4,5,8,10^

This is the first study to compare three different image enhancement systems: professional image enhancement technology, Image1 S and narrow-band imaging. One hundred patients with laryngeal changes were evaluated by three different investigators. The results indicate that the three different systems are comparable.

The prognosis of laryngeal carcinoma varies; it depends, among other factors, on the anatomical location of the tumour, the clinical stage at initial diagnosis and the choice of therapeutic approach.^1,3,13^ Pre-operative diagnostic tools can facilitate the early diagnosis of mucosal lesions. Changes in the vascular pattern can highlight suspicious lesions.

For decades, white light endoscopy and stroboscopy were the ‘gold standard’. Stroboscopy allows evaluation of the mucosal wave and provides information about possible soft tissue invasion. With the development of image enhancement techniques, a supplementary diagnostic tool became available, but, initially, this so-called ‘optical biopsy’ was only possible with the narrow-band imaging system.^1,2,4^

Image1 S and professional image enhancement technology systems have been available for a few years.^3,9^ Narrow-band imaging uses narrow-band light, and the other two systems use a digital software algorithm to visualise vascular changes.

The data from this study show that all three image enhancement systems can reliably diagnose suspicious lesions in the larynx and hypopharynx with high accuracy. The results are comparable to those reported in the literature.^3^ Benign lesions were confirmed in 143 cases with professional image enhancement technology, in 141 cases with narrow-band imaging and in 135 cases with the Image1 S system. Malignancy was detected in 144 cases with professional image enhancement technology, in 145 cases with narrow-band imaging and in 147 cases with the Image1 S system. The higher the Ni classification, the more precise the ‘optical biopsy’. Image enhancement systems allow an oncological assessment which is fundamental given that the detection of malignant lesions is the main focus.

Professional image enhancement technology and narrow-band imaging were applied during a flexible endoscopic procedure, while the Image1 S system was used during rigid endoscopy under general anaesthesia. The transnasal procedure is performed in an upright, sitting, awake patient. With rigid endoscopy in general anaesthesia, it is possible to improve the endoscopic evaluation by suction cleaning of the tissue. Rapid cleaning of the lens is possible without further disturbing the patient or shifting the field of vision. This might explain the higher detection rate of the Image1 S system. However, rigid endoscopy requires general anaesthesia, while flexible endoscopy is easy to perform and does not require sedation.

As the vascular patterns change with malignancy, the rating becomes more distinct. Data analysis shows that a higher Ni laryngeal lesion classification rating has better histological accordance than a lower Ni classification rating. However, the only differentiation between Ni types I and II is the dilation of vessel diameter. Ni type III indicates optical interruption of the vessel integrity, which is sometimes difficult to assess correctly. Thus, secretions or minor endoscopic vision are limited for the lower Ni types, whereas the higher types are more easily detected.

The differentiation of possible malignant changes is the main purpose of image enhancement. The current study data showed that, within the range of Ni types I–III and types IV and V, there was variability between the three different raters. However, there was high agreement in terms of benign and malignant vascular changes. In 95 per cent (professional image enhancement technology), 94 per cent (narrow-band imaging) and 92 per cent (Image1 S) of cases, a minimum of two raters gave the same classification. The sensitivity and specificity for the different image enhancement systems were in the same range as reported in the literature.^1–5^

Another fundamental diagnostic tool in the investigation of premalignant and malignant laryngeal lesions is stroboscopy. The professional image enhancement technology system allows switching between image enhancement and high-definition stroboscopy without major loss of illumination. A malignant process changes the behaviour of the mucosal wave. The mucosal wave and the detection of pathological vessels in pre-operative assessment as well as in post-operative monitoring give primary information about the soft tissue and the functional pattern.

- Image enhancement systems are important diagnostic tools in detecting laryngeal pathologies
- This study compared three different image enhancement systems: professional image enhancement technology, Image1 S and narrow-band imaging
- These image enhancement systems gave comparable results in the detection of laryngeal lesions
- With additional and comparable image enhancement systems, more users can perform image-enhanced endoscopy
- This leads to a broadly available and more precise diagnostic tool, and may help to improve oncological assessment

With two additional image enhancement systems, more users can perform image enhancement endoscopy. This leads to a widely available and more precise diagnostic tool, and can help to improve the oncological assessment. The results of the three systems are comparable to those reported in the literature. There was no significant correlation between system ratings and the three raters’ different experiences with endoscopic image-enhanced evaluation of vascular patterns.

## Conclusion

Since the development of narrow-band light endoscopy, image enhancement has been a fundamental diagnostic tool in laryngology. With the development of Image1 S and professional image enhancement technology, two more image enhancement systems are now available on the market. In contrast to narrow-band imaging, the other two systems use an offline algorithm for image enhancement. The data from this study show that endoscopic classification using the different systems is comparable.

Flexible endoscopy in an awake patient does not have any major disadvantages compared to rigid endoscopy under general anaesthesia. In combination with stroboscopy, image enhancement provides fundamental information about soft tissue changes and laryngeal function. With two additional image enhancement systems, more users can achieve image enhancement endoscopy, which is essential for oncological assessment.
